# The Impact of Transport on Population Health and Health Equity for Māori in Aotearoa New Zealand: A Prospective Burden of Disease Study

**DOI:** 10.3390/ijerph19042032

**Published:** 2022-02-11

**Authors:** Edward Randal, Caroline Shaw, Melissa McLeod, Michael Keall, Alistair Woodward, Anja Mizdrak

**Affiliations:** 1 Department of Public Health, University of Otago, Wellington 6242, New Zealand; caroline.shaw@otago.ac.nz (C.S.); melissa.mcleod@otago.ac.nz (M.M.); michael.keall@otago.ac.nz (M.K.); anja.mizdrak@otago.ac.nz (A.M.); 2 Epidemiology and Biostatistics, The University of Auckland, Private Bag 92019, Auckland 1142, New Zealand; a.woodward@auckland.ac.nz

**Keywords:** transport, health, inequities, Māori, burden of disease

## Abstract

Background: The land transport system influences health via a range of pathways. This study aimed to quantify the amount and distribution of health loss caused by the current land transport system in Aotearoa New Zealand (NZ) through the pathways of road injury, air pollution and physical inactivity. Methods: We used an existing multi-state life table model to estimate the long-term health impacts (in health-adjusted life years (HALYs)) and changes in health system costs of removing road injury and transport related air pollution and increasing physical activity to recommended levels through active transport. Health equity implications were estimated using relative changes in HALYs and life expectancy for Māori and non-Māori. Results: If the NZ resident population alive in 2011 was exposed to no further air pollution from transport, had no road traffic injuries and achieved at least the recommended weekly amount of physical activity through walking and cycling from 2011 onwards, 1.28 (95% UI: 1.11–1.5) million HALYs would be gained and $7.7 (95% UI: 10.2 to 5.6) billion (2011 NZ Dollars) would be saved from the health system over the lifetime of this cohort. Māori would likely gain more healthy years per capita than non-Māori, which would translate to small but important reductions (2–3%) in the present gaps in life expectancy. Conclusion: The current transport system in NZ, like many other car-dominated transport systems, has substantial negative impacts on health, at a similar level to the effects of tobacco and obesity. Transport contributes to health inequity, as Māori bear greater shares of the negative health impacts. Creating a healthier transport system would bring substantial benefits for health, society and the economy.

## 1. Introduction

Transport impacts health in many ways [1,2,3]. Transport provides access to education, employment, healthcare, food and other amenities but car-dominated transport systems contribute to major global health challenges, including climate change, air pollution, obesity and road injury [4,5]. Transport also causes ill-health from noise, environmental degradation, stress and a host of diseases associated with physical inactivity (including type 2 diabetes, cardiovascular disease, dementia and bowel and breast cancers) [6].

We also know that the impacts of transport on health are often inequitably distributed [7,8]. In car-dominated transport systems, those that use the transport system least (often the poor, ethnic minorities, and indigenous populations) frequently bear more than their share of the negative effects on health [7]. In Aotearoa New Zealand (NZ) the government has an obligation, both under Te Tiriti o Waitangi (the treaty between the Crown and Māori–the Indigenous peoples of Aotearoa) and the United Nations Declaration on the Rights of Indigenous Peoples, to protect the interests and rights of Māori and ensure their wellbeing [9,10,11]. Despite this, Māori, particularly young Māori males, have disproportionately high rates of severe or fatal road injury [12], often face difficulties using the transport system [13,14,15], and have disproportionately high rates of diseases associated with physical inactivity (in particular type 2 diabetes and obesity) despite participating in similar levels of physical activity as non-Māori [16]. Māori also have substantially lower life expectancies than non-Māori (73.4 and 77.1 versus 80.9 and 84.4 for males and females, respectively) [17]. The Māori population also has a substantially younger age distribution, with median ages of 25.1 and 27.1 years for males and females, respectively (the national median ages for males and females are 36.4 and 38.5 years) [18].

Many studies have taken a burden of disease approach to quantifying the health impacts of transport [19,20,21,22,23,24,25,26]. These studies have used a variety of scales (e.g., city vs. national) and included a range of transport-related health exposures (e.g., physical activity, air quality, injury, noise and greenspace exposure). Depending on the scope of the study, transport has been estimated to contribute between two and 20 percent of premature health loss [20,21,23,27,28]. In the studies that considered inequity, a disproportionate amount of the health burden was experienced by people of low socioeconomic status and ethnic minorities [21,23]. All of these studies have used a comparative risk assessment methodology to estimate an annual health burden by assuming an immediate health impact from a change in transport-related exposures. To date, no one has accounted for the known time lags in a health response from changes to transport-related exposures. Moreover, despite the substantive impact of land transport on population health, no one has estimated the contribution of land transport to existing (or future) health inequities.

The aim of this study is to quantify the impact of the current land transport system in NZ on future health loss, inequities in health and costs on the health system. To do this we have carried out a prospective burden of disease modelling exercise [29], using a counterfactual scenario of an improved transport system.

## 2. Methods

### 2.1. Scenario Details

A prospective burden of disease study often requires a counterfactual scenario; an ‘alternative future’ to compare against. We modelled the counterfactual scenario of no serious or fatal road injury, no air pollution from transport and adequate physical activity compared to a continuation of the current transportation system. Our modelled scenario was not one of a transport system with no land transport or even no private motorised vehicle use but rather one that quantifies the impact of the current land transport system in NZ on future health loss, inequities in health and costs on the health system. This counterfactual scenario specifically involved setting road injury morbidity and mortality to zero, removing domestic land transport-generated particulate matter (typically 11% of total PM2.5 exposure [30]) and changing physical activity levels so that all people achieve at least the minimum recommended weekly amount of physical activity of at least 150 min per week of moderate to vigorous physical activity (MVPA), equating to 675 MVPA MET mins/week (metabolic equivalent of task minutes per week).

We modelled the scenario as an immediate change of exposure in the base year (2011). Lags in health effects were modelled (e.g., health benefits from physical activity did not occur immediately). This gave an estimate of the total potential improvement to health from the transport system for the 2011 population modelled forward over their lifetime. Changes in each of the three transport-related exposures (physical activity, air pollution and road injury) were also modelled separately to determine the contribution of each exposure to the total change in health.

### 2.2. Health Impact Modelling

We used the Physical Activity and Active Transport Model (PAATM), an existing multi-state life table (MSLT) model developed for the Burden of Disease Epidemiology, Equity & Cost-Effectiveness Programme to estimate the long-term health impacts and changes in health system costs of removing the main negative health exposures from land transport. The conceptual framework and model inputs have been presented elsewhere [30]. Full model details are published in a technical report [31]. Study-specific details are reported below.

The MSLT model uses changes in physical activity, road injury and exposure to fine particulate matter (<2.5 µm diameter) to estimate health effects in terms of health adjusted life years (HALYs), changes in life expectancy and health system costs over the remaining lifespan of the NZ resident population alive in 2011. The diseases included in the model are coronary heart disease (CHD), stroke, type 2 diabetes, lung cancer, colorectal cancer, breast cancer, chronic obstructive pulmonary disease (COPD), lower respiratory tract infection (LRTI), and road injuries. All dose-response relationships come from systematic reviews and meta-analyses, listed in the technical report [31]. Data on physical activity levels, particulate matter exposure, injury and disease morbidity and mortality, as well as health care costs come from NZ-specific estimates. Model inputs and data sources are listed in Table 1. Full details of the parameters, including values and uncertainty distributions used, are published in Mizdrak, Blakely, Cleghorn and Cobiac [31] and Mizdrak, Blakely, Cleghorn and Cobiac [30].

Health impacts were estimated by age, sex and ethnicity (Māori and non-Māori). Health system costs are reported in 2011NZD and converted to 2020 USD using the Consumer Price Index (CPI) and Purchasing Power Parity (PPP) adjustment. Costs are presented for the total population. Changes in health by ethnicity and sex are presented as per capita estimates as well as totals. To provide per capita estimates, total changes in HALYs were divided by the 2011 estimated population of each sex and ethnicity group. Ethnicity was identified as recorded in source datasets. All-cause mortality and morbidity rates under the baseline and counterfactual scenario were used to estimate life expectancy and healthy life expectancy. Māori-specific mortality and morbidity rates were used to estimate changes in HALYs and life expectancy for Māori. Changes in health by ethnicity are also presented in age-standardised Māori: non-Māori ratios of HALYs gained. The Māori population is significantly younger than the non-Māori population. In order to estimate the effect of these differing age structures and to identify differences in health impact not related to age, per capita health changes were age-standardised [32]. We used direct standardisation based on the method set out in Robson, et al. [33]. For simplicity, we used the internal (2011) Māori population as the standard population. Age-specific per capita changes in HALYs for the non-Māori population were applied to the standard population to estimate the changes in health if non-Māori had the same age structure as Māori.

Modelling was carried out in Microsoft Excel (Office 365); 95% Uncertainty intervals (UI) of all estimates were calculated using Monte Carlo analysis with 2000 simulations based on the probabilistic sampling of pre-defined uncertainty distributions for each input parameter. Full details of this process are presented in the technical report [31].

## 3. Results

**Figure 1:**
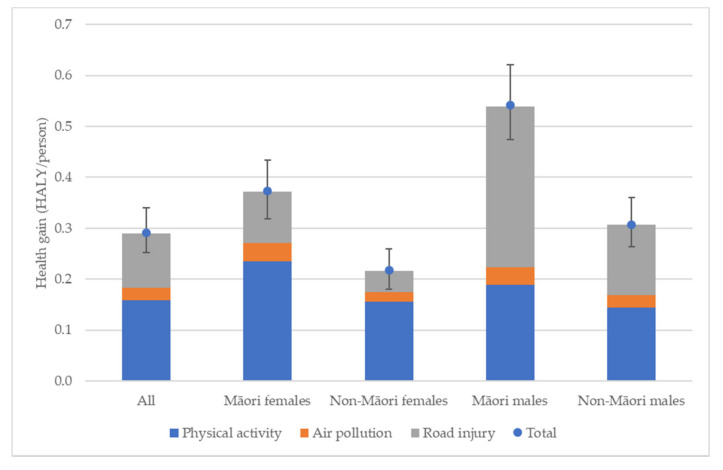
Proportion of health gains by exposure. HALY = Health adjusted life years. Error bars = 95% Uncertainty Interval.

If the New Zealand (NZ) resident population alive in 2011 were exposed to no further air pollution from transport, had no road traffic injuries and achieved at least the recommended weekly amount of physical activity through walking and cycling from 2011 onwards, 1.28 (95% UI: 1.11–1.50) million HALYs would be gained and $7.72 (95% UI: 10.17 to 5.56) billion (in 2011 NZ Dollars-approximately $6 billion 2020 US dollars with purchasing power parity) would be saved from the health system over the lifetime of this cohort (Table 2). The majority of this benefit will come from increases in physical activity. If the physical activity improvements alone were implemented, 58% of the total health gains would be realised; 37% would be realised by solely reducing injury and 8% would be realised by improving air quality (‘All’ column of Figure 1). These proportions are calculated by running each exposure individually and comparing the results with those from the full scenario. The HALYs by exposure, therefore, do not add up to the overall burden because of overlap in health effects between the exposures (particularly air pollution and physical activity both impacting cardiovascular disease).

Per capita, Māori gain more HALYs than non-Māori. Māori females gain approximately 72% more HALYS than non-Māori females (0.373 HALY/person compared to 0.217 HALY/person, respectively). Māori males also gain approximately 76% more HALYS than non-Māori males (0.542 HALY/person compared to 0.307 HALY/person, respectively) (Table 3 and Figure 1). Health gains are strongly patterned by age, with younger population groups gaining more HALYS than older age groups (partly due to the lifetime modelling perspective). After adjusting for the older age structure of the non-Māori population, Māori females gain 46% more HALY/person than non-Māori females and Māori males gain 43% more HALY/person than non-Māori males.

By modelling each exposure separately, we can see the contribution each one makes to overall health gains by ethnicity and gender (Figure 1). As with the total population, physical activity is the largest contributor to health gains for females, with Māori females receiving 63% and non-Māori females 72% of total transport-related health gains from physical activity. However, this trend does not hold for males. Non-Māori males receive approximately equal health gains from physical activity and road injury reduction (47% and 45%, respectively), while Māori males gain substantially more health from road injury reduction than physical activity (58% and 35%, respectively, as a proportion of health gain under this scenario). The proportion of health gains under this scenario that arise from particulate air pollution improvements remain reasonably consistent across all groups ranging from 6% (Māori males) to 10% (Māori females). This is to be expected as ethnicity- and gender-specific air pollution exposures were not available at the time the model was created.

Per capita, health gains by exposure show Māori gain more HALYs per person than non-Māori for all exposures (Table 3), with the greatest relative HALY gains coming from injury reduction with Māori to non-Māori per capita HALY ratios of 2.29 for males and 2.41 for females (1.71 and 1.87, respectively, when age-standardised).

**Figure 2:**
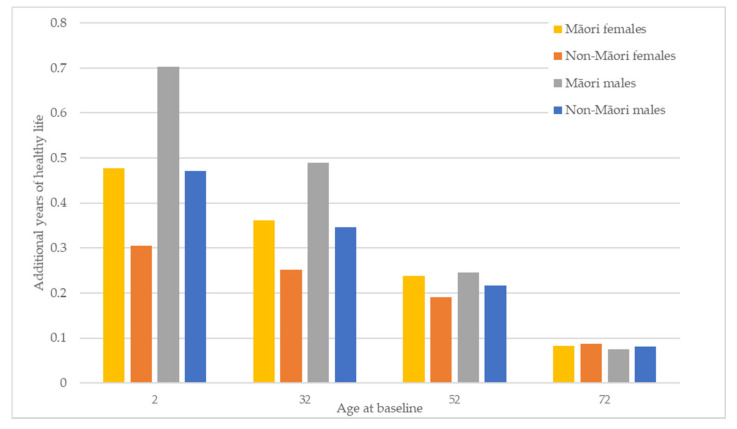
Changes in healthy life expectancy by ethnicity and gender for four age cohorts.

Figure 2 shows how the HALY gains discussed above translate into gains in healthy life expectancy across all groups. Gains in life expectancy are greatest in the youngest cohort (age 2 years). Māori and males see the largest increases in life expectancy, with Māori females gaining more life expectancy than non-Māori females and Māori males gaining more than non-Māori males across all age groups. However, the baseline difference in healthy life expectancy between Māori and non-Māori for the youngest age group is 8.11 and 7.42 years for males and females, respectively. The gain in life expectancy modelled here will reduce this difference to 7.88 and 7.25 years, respectively. This equates to an additional 85 days of healthy life for Māori males and 63 days of healthy life for Māori females compared to the healthy days of life gained by non-Māori and a reduction in the healthy life expectancy gap between Māori and non-Māori of three and two percent for males and females, respectively.

**Figure 3:**
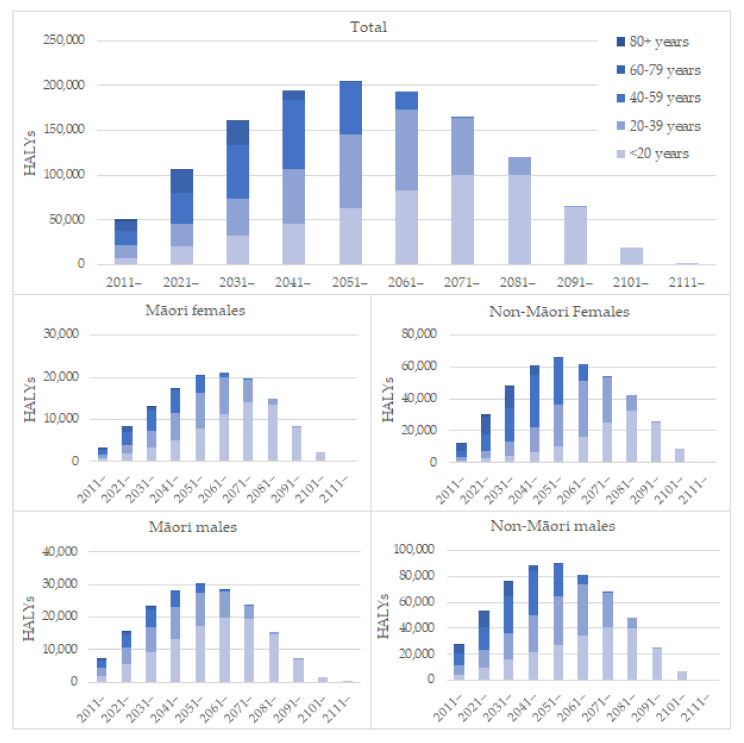
Health gains over time by ethnicity and gender. HALY = Health adjusted life years.

Looking at when these gains are realised (Figure 3), we see most gains for the total population are realised within 40 years of the changes in exposures, with older people benefiting earlier and younger people benefiting as they increase in age. The pattern for Māori shows proportionately larger gains for younger people and relatively small gains for older people compared to the total which reflects the younger population’s age structure. The pattern for non-Māori females shows proportionately larger gains for older people and small gains for younger people, particularly in the earlier years of the modelled period.

**Figure 4:**
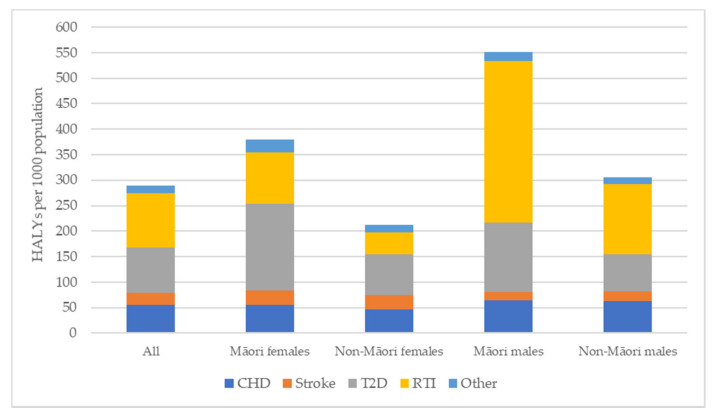
Health gains by disease type. HALY = Health adjusted life years; CHD = Coronary heart disease; T2D = Type-2 Diabetes; RTI = Road traffic injury.

Breaking down the health gains by disease type (Figure 4) shows that for all groups, road injury, type 2 diabetes, CHD and stroke make up the majority of health gains, ranging from 93% (Māori and Non-Māori females) to 97% (Māori males) of total HALYs gained. However, the pattern between these four main diseases varies, with type 2 diabetes being the largest contributor for females, in particular Māori females, and injury being the largest for males. While Māori males gain more HALYs compared to non-Māori males across all the diseases except stroke, road injury reduction is a particularly important contributor for Māori males, accounting for over half the total health gains (57%).

## 4. Discussion

### 4.1. Summary of Findings

The scenario modelled provides an estimate of the potential health that could be gained by addressing the health impact of the current transport system of Aotearoa New Zealand (NZ) for those alive in 2011. For the modelled cohort eliminating all road injury and air pollution caused by land transport and ensuring all people achieve at least the recommended levels of physical activity from active travel would result in substantial health gains and savings to the health system. Addressing transportation would have measurable impacts on health equity. In our model, Māori would gain more HALYs (than non-Māori) with a small reduction in existing inequities in healthy life expectancy. These results can also be framed in terms of opportunity costs: by doing nothing to reconfigure the transport system, this is the amount of health that will be lost from the people alive in 2011 in NZ. Everyone born since 2011 will also lose health and every year that the health impacts of transport are not addressed locks in lost health, health inequity and health costs.

The figures presented here are not directly comparable to other transport-related burden of disease studies due to different modelling methodologies. However, broadly speaking, our findings show a similar pattern of health impact by exposures to other transport health impact models with physical activity making the largest contribution to health [6,39]. Air quality has a smaller effect on health in our study than in transport burden of disease studies of European countries and cities [22,26,28], which is likely due to the lower levels of air pollution in NZ compared to Europe [40]. The per capita burden of road injury in NZ is substantially higher than in Europe and the United States and was seen in this study to be a major cause of health loss for Māori males [20]. Many factors are likely to contribute to these national differences, including differing road conditions, age and safety of the national vehicle fleet, transport policies and other economic, social and environmental influences on road injury risk [41].

### 4.2. Transport’s Role in Health Inequity

Creating a healthy transport system would improve existing health inequities in road injury and diseases associated with air pollution and physical inactivity, using both relative and absolute measures of inequality, and lead to a small, but measurable, improvement in life expectancy for Māori. Māori HALY gains per capita from each of the three pathways were greater than per capita gains for non-Māori. This supports the finding of Lindsay, et al. [42] that the physical activity benefits of strategies to increase active travel will be greater for Māori. However, much of the health gain for Māori males would come from addressing road injury, which predominantly impacts younger people and so has a large effect on years of life lost. Even after age-standardisation, Māori stand to gain substantially more health per capita than non-Māori. Disparities in the health impact of transport have also been highlighted in Bradford, UK, where residents of more ethnically diverse neighbourhoods had the highest risk of premature death from transport-related exposures [23]. Given that transport exposures and the related disease patterns are worse in more deprived areas, we could expect removing the negative health impacts of transport to also reduce socioeconomic health inequities [21].

Much of the modelled health benefit, particularly for non-Māori, accrues at older ages. This reflects the older age structure of the non-Māori population as well as the fact that diseases affected by physical activity (the largest contributor to non-Māori health gains) and air pollution generally impact health later in life. Māori see greater improvements to health at younger ages, as a result of the combined impact of Māori having a younger age structure and being at higher risk of road traffic injury (irrespective of age) [12]. These results show positive potential for improving equity, Māori will benefit earlier from actions to reduce the health impacts of transport—the speed of change in health from improving transport is greater for Māori than non-Māori.

The findings from this modelling show that the current transport system negatively impacts the health of Māori more than non-Māori regardless of age. The precise causes of the disparities in road crash injury for Māori (which are particularly marked for car/van occupants and pedestrians) require further research [12]. However, it is likely contributing factors include differential exposure to environmental hazards (neighbourhood traffic density and speeds for example), along with compounding effects of social disadvantage [43]. These results also indicate issues with structural racism within the design of the transport system that needs to be addressed. There is a lack of both theory and research into how racism is expressed and reinforced through the transport system. However other research has indicated transport reinforces pre-existing disadvantages as a pathway to the criminal justice system for young Māori men, that Māori have lower access to cars and bicycles, and often do not see themselves reflected in healthy transport behaviours, such as cycling [13,14,15,44]. Moreover, policy in this area is lacking; with little, if any, consideration of inequities in policy design and evaluation.

### 4.3. Transport as a Determinant of Health

These results also allow us to look at the role of the transport system as a determinant of population health alongside other important health determinants. Removing transport-related air pollution and road injury and increasing physical activity to recommended levels through walking and cycling will have a similar (or larger) effect on overall life expectancy as eradicating tobacco or obesity [29,45].

Transport is arguably a more complex policy issue than tobacco or obesity as the benefits of mobility are substantial. The policy challenge is to decouple the benefits that mobility confers to individuals and society from the adverse and inequitable impacts of the current system that enables this mobility. Importantly the current policy paradigm that delivers these benefits (known variously as automobility or car dependence) represents only one version of mobility [46]. There is a wide range of alternatives from re-designing urban form to promoting shared transport solutions that could re-shape mobility. The policy lessons from tobacco control (and public health more generally) are likely to be helpful to understand the most effective approaches to re-shaping the transport system into one that promotes health and reduces inequity [47,48,49]. This reshaping will require shifting transport policy away from being an economic and technical issue to being a central part of social policy, bringing with it the duties and responsibilities that social policy requires [8].

We have not suggested specific transport or urban planning solutions to achieve the scenario modelled. There are many ways we could realise this scenario, but all will involve coordinated effort from transport planners, urban designers and land-use planners to create cities that encourage active and public transport and that are compact and polycentric to reduce the need to drive (whilst still allowing for those who need to use a car), to create roads that provide for vulnerable users and create safe environments for all people, and design policies that restrict behaviours or choices that impose a substantial risk to other people or restrict other people’s freedoms and capabilities (e.g., restricting unsafe, large or polluting vehicles). However, the health and equity gains will be very sensitive to the way in which such a transport system was achieved. Therefore, specific solutions to achieve a healthier transport system would be a matter for the society affected to consider. This process should pay particular attention to those most disadvantaged and take into account the capabilities and outcomes those people value [8].

### 4.4. Strengths and Limitations

To our knowledge, this is the first study to quantify the contribution of the land transport system to health inequities in NZ. It is also the first study to examine the prospective burden of disease from the current transport system. The forward-looking perspective of the lifetable modelling approach is a key strength over typical comparative risk assessments that allows us to view the impact of the current transport system at any year in the future, rather than simply comparing two static representations of possible health scenarios. Using a lifetable modelling approach has allowed us to factor in delays in health effects from changes to transport patterns, more accurately quantify the long-term health effects and observe the pattern of health effects, including effects on life expectancy, future disease rates and future health system costs, over the life of an entire population. This produces a clearer picture of the likely health effects of policies to address the negative health impacts and health inequities of transport [30,31].

However, one of the limitations of our approach is that the results produced are likely to be significant underestimates of the true burden of disease from transport. This model contains only a subset of the pathways between transport and health and the health outcomes associated with the pathways. For example, PAATM does not include the health effects of changes in obesity, dementia, or depression that result from changes in physical activity and includes a limited list of cancers affected by physical activity. Neither does it include the health effects of transport sourced NO_2_ pollution and noise exposure. The health impacts of air pollution in NZ are currently being revised and the burden of disease is likely to increase substantially from those used in this model, particularly once the contribution of NO_2_ is included [40]. Moreover, noise pollution has been found to be a substantial contributor to transport-related burden of disease in some jurisdictions. For example, studies in Barcelona and Warsaw estimated the health effects of traffic noise were greater than road injury and air pollution [22,26]. However, a health impact assessment of road transport in NZ estimated the health impact of traffic noise to be substantially less than road injury and air pollution (917 DALYs attributable to noise compared to 21,244 and 4449 DALYs attributable to road injury and air pollution, respectively) [20]. Ongoing model development aims to address many of these gaps in the future.

Another possible source of underestimation is the inability to account for feedback cycles that could be expected to occur if we reoriented the transport system. We have modelled impacts through disease incidence, but it is plausible that a better functioning transport system may also lead to reductions in case fatality from diseases through improved healthcare access and earlier diagnosis and treatment.

It is also worth noting that this modelling exercise was carried out specifically for NZ with gender- and ethnicity-specific disease rates of this high-income, highly car dependent country. While we would expect similar results for other highly car dependent countries with similar baseline disease rates, results will be country-specific and the findings presented here may not be generalisable to other jurisdictions.

The scenario we have presented is ambitious within the current transport paradigm. However, some jurisdictions have achieved components of this modelled scenario demonstrating that the scenario is achievable. For example, although caution must be exercised in drawing comparisons across countries due to differences in population, urbanicity, and other factors, average active travel in the Netherlands is above the level we have modelled [50]. Similarly, Oslo, Norway, is close to achieving zero road deaths with only one fatality recorded in 2019, suggesting this goal is achievable at least for the large urban centres of NZ if not the entire country [51]. Moreover, the modelled scenario is a worthy goal for policy makers to aim for. It is also worth noting that other eradication scenarios have also been seen as impossible at first but have changed over time (for example NZ is currently consulting on how to become tobacco free [52]).

## 5. Conclusions

Historical and current policy choices in transport matter enormously for health and health equity. The results presented here emphasise that the current transport system in Aotearoa New Zealand, like many other car-dominated transport systems, has substantial negative impacts on health. This system is also one of the many contributors to health inequity, with Māori bearing the greatest share of these negative health impacts from road injury and physical inactivity (and likely other unmodelled health exposures). The large contribution of physical inactivity to the transport-related health burden highlights that finding equitable and sustainable solutions to these health problems will require a systematic approach from policymakers. All of the pathways from transport to health need to be considered, and the traditional transport agency’s focus on mobility and road safety must change to a broader view of transport health and safety. On the basis of our modelling, we conclude that achievable changes in the transport system can bring substantial benefits for health, society and the economy.

## Figures and Tables

**Table 1:** Model inputs and data sources. NZ = New Zealand; MVPA = moderate to vigorous physical activity; MET = metabolic equivalent of task.

Model Input	Ethnicity Specific	Source
Population estimates by age, sex and ethnicity	Yes	Statistics NZ 2011 Estimated population count. Values used published in Burden of Disease Epidemiology Equity and Cost-Effectiveness Programme [34]
Physical activity (MVPA-METmins/week)	Yes	Distribution calculated from NZ Health Survey 2011/12 using MET values from Ainsworth, et al. [35]. Values used published in Mizdrak, Blakely, Cleghorn and Cobiac [31]
Air pollution	No	Brauer, et al. [36]. Values used published in Mizdrak, Blakely, Cleghorn and Cobiac [31]
Injury incidence and mortality	Yes	Derived from Global Burden of Disease Study [37], Ministry of Health [38], and Ministry of Health National Collections. Values used published in Mizdrak, Blakely, Cleghorn and Cobiac [31]
Disease incidence, prevalence and mortality	Yes	Ministry of Health [38], Ministry of Health National Collections. Values used published in Burden of Disease Epidemiology Equity and Cost-Effectiveness Programme [34]
All-cause mortality rates	Yes	Statistics NZ period Life Table 2010, Table 2011 and Table 2012. Values used published in Burden of Disease Epidemiology Equity and Cost-Effectiveness Programme [34]
Morbidity rates	Yes	Global Burden of Disease Study [37], Ministry of Health [38]. Values used published in Burden of Disease Epidemiology Equity and Cost-Effectiveness Programme [34]
Health system costs	No	Analysis of Ministry of Health National Collections. Values used published in Mizdrak, Blakely, Cleghorn and Cobiac [31]

**Table 2:** Health adjusted life years and health system costs. HALY = Health adjusted life years; NZD = New Zealand dollars.

		Overall Burden (95% Uncertainty Interval)	Physical Activity Only (95% Uncertainty Interval)	Air Pollution Only (95% Uncertainty Interval)	Road Injury Only (95% Uncertainty Interval)
HALYs (thousands)	All	1280 (1110 to 1500)	702 (583 to 846)	106 (67.5 to 155)	472 (386 to 562)
	Māori females	128 (109 to 149)	80.6 (65.3 to 96.4)	12.4 (8 to 19.1)	34.7 (28.4 to 41.3)
	Non-Māori females	412 (343 to 493)	295 (236 to 361)	37.1 (23.2 to 54.2)	79.7 (60.4 to 98.6)
	Māori males	179 (157 to 206)	62.7 (51.4 to 75.5)	11.1 (7.2 to 17)	105 (88.5 to 122)
	Non-Māori males	563 (483 to 662)	264 (212 to 327)	45.2 (28 to 65.7)	253 (205 to 301)
Health system costs (NZD2011 billions)	Total	−7.72 (−10.17 to −5.56)	−8.2 (−10.36 to −6.45)	−0.7 (−1.13 to −0.36)	1.2 (0.17 to 2.31)

**Table 3:** Per capita Health Adjusted Life Years (HALYs).

	Total (95% Uncertainty Interval)		Physical Activity Only (95% Uncertainty Interval)		Air Pollution Only (95% Uncertainty Interval)		Road Injury Only (95% Uncertainty Interval)	
	Raw	Age-Standardised	Raw	Age-Standardised	Raw	Age-Standardised	Raw	Age-Standardised
All (HALY/person)	0.29 (0.25 to 0.34)	0.39 (0.34 to 0.45)	0.16 (0.13 to 0.19)	0.19 (0.16 to 0.23)	0.02 (0.02 to 0.03)	0.03 (0.02 to 0.04)	0.11 (0.09 to 0.13)	0.16 (0.14 to 0.19)
All ratio (Māori: non-Māori)	1.74 (1.65 to 1.85)	1.45 (1.38 to 1.52)	1.42 (1.35 to 1.48)	1.25 (1.19 to 1.30)	1.58 (1.28 to 1.67)	1.33 (1.07 to 1.40)	2.32 (2.1 to 2.64)	1.75 (1.59 to 1.95)
Māori females (HALY/person)	0.37 (0.32 to 0.43)	0.37 (0.32 to 0.43)	0.24 (0.19 to 0.28)	0.24 (0.19 to 0.28)	0.04 (0.02 to 0.05)	0.04 (0.02 to 0.05)	0.10 (0.08 to 0.12)	0.10 (0.08 to 0.12)
Non-Māori females (HALY/person)	0.22 (0.18 to 0.26)	0.26 (0.21 to 0.30)	0.16 (0.12 to 0.19)	0.18 (0.14 to 0.22)	0.02 (0.01 to 0.03)	0.02 (0.01 to 0.03)	0.04 (0.03 to 0.05)	0.05 (0.04 to 0.07)
Female ratio (Māori: non-Māori)	1.72 (1.64 to 1.81)	1.46 (1.40 to 1.53)	1.51 (1.44 to 1.58)	1.33 (1.27 to 1.38)	1.85 (1.50 to 1.98)	1.55 (1.25 to 1.66)	2.41 (2.15 to 2.81)	1.87 (1.68 to 2.14)
Māori males (HALY/person)	0.54 (0.47 to 0.62)	0.54 (0.47 to 0.62)	0.19 (0.16 to 0.23)	0.19 (0.16 to 0.23)	0.03 (0.02 to 0.05)	0.03 (0.02 to 0.05)	0.32 (0.27 to 0.37)	0.32 (0.27 to 0.37)
Non-Māori males (HALY/person)	0.31 (0.26 to 0.36)	0.38 (0.33 to 0.44)	0.14 (0.12 to 0.18)	0.16 (0.13 to 0.20)	0.02 (0.02 to 0.04)	0.03 (0.02 to 0.04)	0.14 (0.11 to 0.16)	0.19 (0.15 to 0.22)
Male ratio (Māori: non-Māori)	1.76 (1.66 to 1.89)	1.43 (1.36 to 1.52)	1.32 (1.24 to 1.39)	1.16 (1.10 to 1.22)	1.36 (1.09 to 1.42)	1.15 (0.92 to 1.20)	2.29 (2.08 to 2.59)	1.71 (1.57 to 1.90)

## Data Availability

All health and demographic data used in this study are available in the PAATM Technical Report. Access to the anonymised and de-identified Household Travel Survey dataset was obtained under a confidentiality deed from the New Zealand Ministry of Transport. None of the authors had access to identifying information about the individuals included in the survey. Researchers wishing to obtain access to the Household Travel Survey should contact the Ministry of Transport at travelsurvey@transport.govt.nz. Conditions of access include (but are not limited to) agreement to strict privacy protocols and the signing of a confidentiality deed. More information about the travel survey is available at www.transport.govt.nz/travelsurvey/.

## References

[1] Woodcock J., Banister D., Edwards P., Prentice A.M., Roberts I. (2007). Energy and Transport. Lancet.

[2] Van Schalkwyk M.C.I., Mindell J.S. (2018). Current Issues in the Impacts of Transport on Health. Br. Med. Bull..

[3] Glazener A., Sanchez K., Ramani T., Zietsman J., Nieuwenhuijsen M.J., Mindell J.S., Fox M., Khreis H. (2021). Fourteen pathways between urban transportation and health: A conceptual model and literature review. J. Transp. Health.

[4] Swinburn B.A., Vivica I., Kraak S.A., Vincent J., Atkins P.I., Baker J.R., Bogard H.B., Calvillo A., De Schutter O., Devarajan R. (2019). The Global Syndemic of Obesity, Undernutrition, and Climate Change: The Lancet Commission Report. Lancet.

[5] Tranter P.J. (2010). Speed Kills: The Complex Links between Transport, Lack of Time and Urban Health. J. Urban Health Bull. N. Y. Acad. Med..

[6] Woodcock J., Edwards P., Tonne C., Armstrong B.G., Ashiru O., Banister D., Beevers S., Chalabi Z., Chowdhury Z., Cohen A. (2009). Public Health Benefits of Strategies to Reduce Greenhouse-Gas Emissions: Urban Land Transport. Lancet.

[7] Banister D. (2018). Inequality in Transport.

[8] Randal E., Shaw C., Woodward A., Howden-Chapman P., Macmillan A., Hosking J., Chapman R., Waa A.M., Keall M. (2020). Fairness in Transport Policy: A New Approach to Applying Distributive Justice Theories. Sustainability.

[9] Royal Commission on Social Policy (1988). The April Report Colume 2: Future Directions.

[10] Human Rights Commission (2010). Human Rights Commission. Human Rights and the Treaty of Waitangi. Human Rights in New Zealand 2010.

[11] United Nations General Assembly (2007). Resolution 61/295 United Nations Declaration on the Rights of Indigenous Peoples.

[12] Hosking J., Ameratunga S., Exeter D., Stewart J., Bell A. (2013). Ethnic, Socioeconomic and Geographical Inequalities in Road Traffic Injury Rates in the Auckland Region. Aust. N. Z. J. Public Health.

[13] Russell M., Davies C., Wild K., Shaw C. (2021). Pedalling Towards Equity: Exploring Women’s Cycling in a New Zealand City. J. Transp. Geogr..

[14] Jones R., Kidd B., Wild K., Woodward A. (2020). Cycling Amongst Māori: Patterns, Influences and Opportunities. N. Z. Geogr..

[15] Raerino K., Macmillan A.K., Jones R.G. (2013). Indigenous Māori Perspectives on Urban Transport Patterns Linked to Health and Wellbeing. Health Place.

[16] Ministry of Health Annual Data Explorer 2019/20: New Zealand Health Survey [Data File]. https://minhealthnz.shinyapps.io/nz-health-survey-2019-20-annual-data-explorer/.

[17] Statistics New Zealand (2021). National and Subnational Period Life Tables: 2017–2019.

[18] Statistics New Zealand Māori Population Estimates: At 30 June 2020. https://www.stats.govt.nz/information-releases/maori-population-estimates-at-30-june-2020.

[19] Bhalla K., Shotten M., Cohen A., Brauer M., Shahraz S., Burnett R., Leach-Kemon K., Freedman G., Murray J.L.C. (2014). Transport for Health: The Global Burden of Disease from Motorized Road Transport (English).

[20] Briggs D., Mason K., Borman B. (2016). Rapid Assessment of Environmental Health Impacts for Policy Support: The Example of Road Transport in New Zealand. Int. J. Environ. Res. Public Health.

[21] Iungman T., Khomenko S., Nieuwenhuijsen M., Barboza E.P., Ambròs A., Padilla C., Mueller N. (2021). The Impact of Urban and Transport Planning on Health: Assessment of the Attributable Mortality Burden in Madrid and Barcelona and Its Distribution by Socioeconomic Status. Environ. Res..

[22] Mueller N., Rojas-Rueda D., Basagaña X., Cirach M., Cole-Hunter T., Dadvand P., Donaire-Gonzalez D., Foraster M., Gascon M., Martinez D. (2017). Health Impacts Related to Urban and Transport Planning: A Burden of Disease Assessment. Environ. Int..

[23] Mueller N., Rojas-Rueda D., Khreis H., Cirach M., Milà C., Espinosa A., Foraster M., McEachan R.R.C., Kelly B., Wright J. (2018). Socioeconomic Inequalities in Urban and Transport Planning Related Exposures and Mortality: A Health Impact Assessment Study for Bradford, UK. Environ. Int..

[24] Sohrabi S., Khreis H. (2020). Burden of Disease from Transportation Noise and Motor Vehicle Crashes: Analysis of Data from Houston, Texas. Environ. Int..

[25] Sohrabi S., Zietsman J., Khreis H. (2020). Burden of Disease Assessment of Ambient Air Pollution and Premature Mortality in Urban Areas: The Role of Socioeconomic Status and Transportation. Int. J. Environ. Res. Public Health.

[26] Tainio M. (2015). Burden of Disease Caused by Local Transport in Warsaw, Poland. J. Transp. Health.

[27] Mueller N., Rojas-Rueda D., Basagaña X., Cirach M., Cole-Hunter T., Dadvand P., Donaire-Gonzalez D., Foraster M., Gascon M., Martinez D. (2017). Urban and Transport Planning Related Exposures and Mortality: A Health Impact Assessment for Cities. Environ. Health Perspect..

[28] Khomenko S., Nieuwenhuijsen M., Ambròs A., Wegener S., Mueller N. (2020). Is a Liveable City a Healthy City? Health Impacts of Urban and Transport Planning in Vienna, Austria. Environ. Res..

[29] Blakely T., Cleghorn C., van der Deen F.P., Cobiac L.J., Mizdrak A., Mackenbach J.P., Woodward A., van Baal P., Wilson N. (2020). Prospective Impact of Tobacco Eradication and Overweight and Obesity Eradication on Future Morbidity and Health-Adjusted Life Expectancy: Simulation Study. J. Epidemiol. Community Health.

[30] Mizdrak A., Blakely T., Cleghorn C.L., Cobiac L. (2019). Potential of Active Transport to Improve Health, Reduce Healthcare Costs, and Reduce Greenhouse Gas Emissions: A Modelling Study. PLoS ONE.

[31] Mizdrak A., Blakely T., Cleghorn C.L., Cobiac L. (2018). Technical Report for BODE^3^ Active Transport and Physical Activity Model.

[32] Ministry of Health (2018). Position Paper on Māori Health Analytics—Age Standardisation.

[33] Robson B., Purdie G., Cram F., Simmonds S. (2007). Age Standardisation—An Indigenous Standard?. Emerg. Themes Epidemiol..

[34] Burden of Disease Epidemiology Equity and Cost-Effectiveness Programme (2019). Disease Inputs Used for Multi-State Life Table Modelling (Version 1.0).

[35] Ainsworth B.E., Haskell W.L., Whitt M.C., Irwin M.L., Swartz A.M., Strath S.J., O’Brien W.L., Bassett D.R., Schmitz K.H., Emplaincourt P.O. (2000). Compendium of Physical Activities: An Update of Activity Codes and Met Intensities. Med. Sci. Sports Exerc..

[36] Brauer M., Freedman G., Frostad J., Van Donkelaar A., Martin R., Dentener F., Van Dingenen R., Estep K., Amini H., Apte J. (2016). Ambient Air Pollution Exposure Estimation for the Global Burden of Disease 2013. Environ. Sci. Technol..

[37] Global Burden of Disease Study (2016). Global Burden of Disease Study 2015 Results.

[38] Ministry of Health (2016). Health Loss in New Zealand: A Report from the New Zealand Burden of Diseases, Injuries and Risk Factors Study, 2006–2013.

[39] de Sá T.H., Tainio M., Goodman A., Edwards P., Haines A., Gouveia N., Monteiro C., Woodcock J. (2017). Health Impact Modelling of Different Travel Patterns on Physical Activity, Air Pollution and Road Injuries for São Paulo, Brazil. Environ. Int..

[40] Hales S., Atkinson J., Metcalfe J., Kuschel G., Woodward A. (2021). Long Term Exposure to Air Pollution, Mortality and Morbidity in New Zealand: Cohort Study. Sci. Total Environ..

[41] Peden M., Scurfield R., Sleet D., Mathers C., Jarawan E., Hyder A.A., Mohan D., Hyder A.A., Jarawan E. (2004). World Report on Road Traffic Injury Prevention.

[42] Lindsay G., Macmillan A., Woodward A. (2011). Moving Urban Trips from Cars to Bicycles: Impact on Health and Emissions. Aust. N. Z. J. Public Health.

[43] Diderichsen F., Andersen I., Manuel C., Andersen A.-M.N., Bach E., Baadsgaard M., Brønnum-Hansen H., Hansen F.K., Jeune B., Jørgensen T. (2012). Health Inequality—Determinants and Policies. Scand. J. Public Health.

[44] Shaw C., Russell M., Keall M., MacBride-Stewart S., Wild K., Reeves D., Bentley R., Woodward A. (2020). Beyond the Bicycle: Seeing the Context of the Gender Gap in Cycling. J. Transp. Health.

[45] van der Deen F.S., Wilson N., Cleghorn C.L., Kvizhinadze G., Cobiac L.J., Nghiem N., Blakely T. (2018). Impact of Five Tobacco Endgame Strategies on Future Smoking Prevalence, Population Health and Health System Costs: Two Modelling Studies to Inform the Tobacco Endgame. Tob. Control.

[46] Mattioli G., Roberts C., Steinberger J.K., Brown A. (2020). The Political Economy of Car Dependence: A Systems of Provision Approach. Energy Res. Soc. Sci..

[47] Woodcock J., Aldred R. (2008). Cars, Corporations, and Commodities: Consequences for the Social Determinants of Health. Emerg. Themes Epidemiol..

[48] Mindell J. (2001). Lessons from Tobacco Control for Advocates of Healthy Transport. J. Public Health Med..

[49] Douglas M.J., Watkins S.J., Gorman D.R., Higgins M. (2011). Are Cars the New Tobacco?. J. Public Health.

[50] Fishman E., Böcker L., Helbich M. (2015). Adult Active Transport in the Netherlands: An Analysis of Its Contribution to Physical Activity Requirements. PLoS ONE.

[51] Hartmann A., Abel S. (2020). How Oslo Achieved Zero Pedestrian and Bicycle Fatalities in 2019, and How Other Cities Can Apply What Worked. Inst. Transp. Eng. ITE J..

[52] Ministry of Health Smokefree Aotearoa 2025. https://www.health.govt.nz/our-work/preventative-health-wellness/tobacco-control/smokefree-aotearoa-2025.

